# Molecular diagnostics in odontogenic tumors

**DOI:** 10.1007/s00292-022-01152-7

**Published:** 2022-11-15

**Authors:** Reinhard Buettner, Sibel Elif Gültekin

**Affiliations:** 1 Institute of Pathology, Medical Faculty, University of Cologne, Cologne, Germany; 2 Dept of Oral Pathology, Faculty of Dentistry, Gazi University, Ankara, Turkey

**Keywords:** Odontogenic tumors, Ameloblastoma, Oncogene mutation, Molecular classification, Neoadjuvant therapy, Odontogene Tumoren, Ameloblastom, Onkogene Mutationen, Molekulare Klassifikation, Neoadjuvante Therapie

## Abstract

**Background:**

Odontogenic tumors (OTs) are rare, with an estimated incidence rate of less than 0.5 cases per 100,000 per year. The causes of OTs remain unclear. Nonetheless, the majority of OTs seem to arise de novo, without an apparent causative factor. Although the etiopathogenesis of most OTs remains unclear, there have been some recent advances in understanding the genetic basis relating to specific histologies and clinical features. Molecular analyses performed by different techniques, including Sanger sequencing, next-generation sequencing, and allele-specific PCR, have uncovered mutations in genes related to the oncogenic MAPK/ERK signaling pathway. Genetic mutations in these pathway genes have been reported in epithelial and mixed OTs, in addition to odontogenic carcinomas and sarcomas. Notably, B‑RAF proto-oncogene serine/threonine kinase (*BRAF*) and KRAS proto-oncogene GTPase (*KRAS*) pathogenic mutations have been reported in a high proportion of ameloblastoma and ameloblastoma-related tumors and adenomatoid odontogenic tumors, respectively.

**Objective:**

To discuss how molecular profiling aids in diagnostic classification of odontogenic tumors.

**Conclusion:**

Molecular profiling of odontogenic tumors helps to identify patients for neoadjuvant therapies and reduces postoperative morbidity

## Abstract (de)

**Hintergrund:**

Odontogene Tumoren (OT) sind seltene Tumoren, und die geschätzte Inzidenzrate liegt bei weniger als 0,5 Fällen pro 100.000 pro Jahr. Die Ursachen für OT sind nach wie vor unklar. Die meisten OT scheinen jedoch de novo zu entstehen, ohne dass es einen offensichtlichen ursächlichen Faktor gibt. Obwohl die Ätiopathogenese der meisten odontogenen Tumoren nach wie vor unklar ist, gab es in jüngster Zeit einige Fortschritte beim Verständnis der genetischen Grundlagen bestimmter odontogener Tumoren. Molekulare Analysen mit verschiedenen Techniken, darunter Sanger-Sequenzierung, Next-Generation-Sequenzierung und allelspezifische Polymerasekettenreaktion (PCR), haben Mutationen in Genen aufgedeckt, die mit dem onkogenen MAPK/ERK-Signalweg in odontogenen Tumoren in Verbindung stehen. Genetische Mutationen in diesen Signalweg-Genen wurden bei epithelialen und gemischten odontogenen Tumoren sowie bei odontogenen Karzinomen und Sarkomen festgestellt. Insbesondere die Protoonkogene *BRAF*, das für die Serin-Threonin-Kinase B‑RAF kodiert, und *KRAS*, das für die GTPase KRAS kodiert, wurden in einem hohen Anteil von Ameloblastomen und mit Ameloblastomen verwandten Tumoren bzw. adenomatoiden odontogenen Tumoren nachgewiesen.

**Ziel:**

Erörtert wird die Frage, wie die molekulare Typisierung bei der diagnostischen Klassifizierung von odontogenen Tumoren hilft.

**Schlussfolgerung:**

Die molekulare Typisierung bei odontogenen Tumoren hilft bei der Identifizierung von Patienten für neoadjuvante Therapien, präzisiert die histopathologische Klassifikation und vermindert postoperative Morbidität.

Odontogenic tumors (OTs) comprise a group of heterogeneous lesions ranging from hamartomatous lesions to malignant neoplasms with different behavior, histology, and even different geographical distribution [1]. The etiopathogenesis of most OTs remains unclear; however, there have been some recent advances in understanding the genetic basis of specific OTs [2]. Detection of genetic factors that are involved in the molecular pathogenesis of OTs helps us in targeted therapy [3]. Herein, we highlight the molecular profiling of OTs and provide evidence for the clinical utility of targeted therapies.

## Odontogenesis and odontogenic tumors

Tooth development (odontogenesis) is initiated by interactions between epithelial and mesenchymal cells derived from the ectoderm of the first branchial arch and the ectomesenchyme of the neural crest. Odontogenesis involves several morphologically distinct stages. Reciprocal signaling between epithelium and ectomesenchyme guides the process of tooth embryonic development, which is fully dependent on Wnt, BMP, FGF, Shh, and Eda signals [4]. The pathogenesis of odontogenic tumors is associated with alterations in components of signaling pathways. For instance, studies in the last decade have described pathogenic mutations in mitogen-activated protein kinases/extracellular signal-regulated kinases (MAPK/ERK) pathway cascade components in benign and malignant odontogenic tumors [2, 5].

## Benign odontogenic tumors

The OT classification is mainly divided into two categories, based on biological behavior, as malignant and benign. Benign tumors are classified into three major categories according to their histogenetic origin: epithelial, mesenchymal, and mixed types [2]. OTs may pose both diagnostic and prognostic challenges due to overlapping histology and a high propensity for local recurrence, even though they are considered benign [1].

### Epithelial tumors

#### Ameloblastoma

**Fig. 1:**
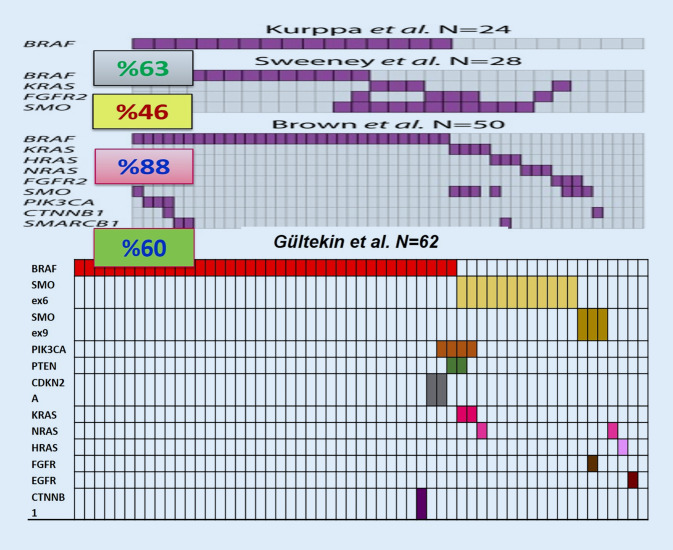
Genomic alterations in ameloblastoma

Ameloblastoma (AM) is the most common benign epithelial odontogenic tumor, representing approximately 1% of all oral tumors and about 9 to 11% of all odontogenic tumors [1]. The tumors are classified into four groups: conventional, unicystic, extraosseous/peripheral, and metastasizing variants. In the recent World Health Organization (WHO) classification, adenoid ameloblastoma (AdAM) is a newly recognized entity separate from the AM group of tumors [2]. Although AMs are known as locally aggressive tumors with high recurrence rates, unicystic ameloblastoma (UAM) shows an indolent course different from the other variants. This unpredictable and distinct biological behavior of AMs has lent them priority to trigger molecular studies on understanding their pathogenesis. In the past decade, oncogenic mutations were discovered which constitutively activate signal transduction pathways relating to developmental stages of odontogenesis, including the mitogen-activated protein kinase (MAPK) and hedgehog pathways [6–8]. Advanced next-generation sequencing (NGS) analyses identified the high frequency of *BRAF* V600E and *SMO* L412F mutations in all types of ameloblastoma [5–8]. This is followed by *KRAS* (mostly p.G12R), *NRAS, HRAS, FGFR2*, and mutations reported in a few *BRAF* wildtype cases ([7–9]; Fig. 1). Additionally, we reported *EGFR* mutations and the presence of other gene mutations, including somatic mutations in *KRAS, PIK3CA, PTEN, FGFR, CDKN2A*, and *CTNNB1* on the background of either *BRAF* or *SMO* mutation-positive ameloblastomas, occurring exclusively in conventional AMs [9].

In line with reports about other neoplasms that harbor a malignant counterpart, the frequency of the *BRAF* p.V600E mutation is higher in ameloblastoma (64% in conventional, 81% in unicystic, and 63% in peripheral) than in ameloblastic carcinoma (35%) [5]. As both conventional AM and UAM have been found to harbor *BRAF* p.V600E mutations, aggressive and destructive tumors could be candidates for BRAF-targeted therapy that has the potential to reduce tumor size and ultimately enable a conservative surgical procedure. Preliminary data of biological treatment show effectiveness in selected cases [2, 3].

#### Adenoid ameloblastoma

Adenoid ameloblastoma (AdAM) is a newly recognized entity separate from the AM group of tumors. AdAM is characterized by an aggressive biological behavior with local infiltration, and the recurrence rate is high (45.5–70%). *BRAF* p.V600E mutations, usually identified in AM/UAM, are absent in AdAM. Whether AdAM is a unique standalone tumor or a histologic variant of AM requires further investigation [2].

#### Adenomatoid odontogenic tumor

**Fig. 2:**
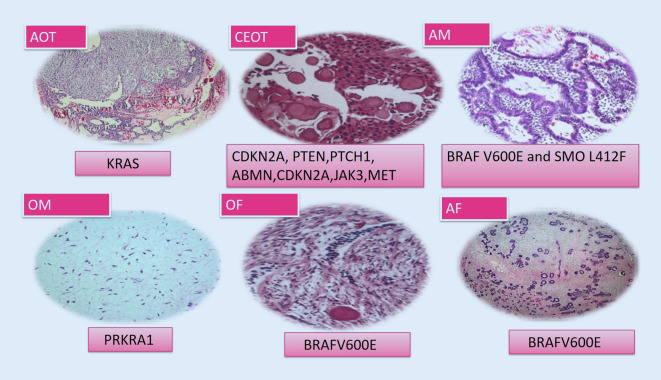
Benign odontogenic tumors and gene mutations

Adenomatoid odontogenic tumor (AOT) manifests clinically as a slow and self-limiting growth which does not require an aggressive surgical approach [1]. AOTs are characterized by frequent *KRAS* codon 12 (either p.G12V or p.G12R, and in a single case p.G12D) driver mutations, which occur in approximately 70% of cases [2, 5, 10]. Although they have not been connected to their clinicopathological features, molecular profiling is important for the differential diagnosis of this tumor from other lesions such as AdAM, adenomatoid odontogenic hamartoma, and adenomatoid dentinoma ([2, 5]; Fig. 2).

#### Calcifying epithelial odontogenic tumor

Calcifying epithelial odontogenic tumor (CEOT) is recognized to have three histopathological subtypes: clear cell, cystic/microcystic, and non-calcified/Langerhans cell rich. Mutations in tumor suppressor genes (*PTEN, CDKN2A, PTCH1*) and oncogenes (*JAK3, MET*) have been identified in CEOT; however, so far, these do not contribute to clinical properties or treatment decisions ([2, 5]; Fig. 2).

### Mixed tumors

#### Odontoma

Odontoma is the most common odontogenic tumor and is composed of mesenchymal and epithelial components of the tooth [1]. WNT/beta-catenin pathway activation in embryonic SOX-2-positive dental stem cells can drive odontoma formation [2]. Ameloblastic fibrodentinoma (AFD) and ameloblastic fibroodontoma (AFO) are classified as developing odontomas, although the prevalence of *BRAF* p.V600E mutations in AFD and AFO is similar to ameloblastic fibroma (AF) but differs from odontoma, which lacks *BRAF* p.V600E mutations [11].

#### Ameloblastic fibroma

Ameloblastic fibroma (AF) is a rare benign odontogenic tumor with the potential for recurrence and malignant transformation to ameloblastic fibrosarcoma ([1]; Fig. 2). AFs are characterized by *BRAF* p.V600E mutations, like other ameloblastic tumors [2, 10, 11]. Early developing stages of odontomas may be comprised of soft tissue closely resembling dental papilla, with prominent epithelial strands and limited or no evidence of dental hard tissue induction. These features overlap with ameloblastic fibroma (AF), sometimes causing a problem differentiating between them. The differentiation between early odontoma and AF is important to avoid unnecessary potentially destructive surgery [1, 2]. Thus, detection of *BRAF* p.V600E mutations is important for differential diagnosis.

### Mesenchymal tumors

#### Odontogenic myxoma

Odontogenic myxoma (OM) is a rare odontogenic tumor that arises from odontogenic ectomesenchyme. The tumor often behaves in a locally aggressive and infiltrating fashion, with a 25% recurrence rate [1]. Activating mutations in the MAPK/ERK signaling pathway have been identified in this tumor and may serve as targets for pharmacologic therapy.

#### Cemento-ossifying fibroma

Cemento-ossifying fibroma (COsF) became an integral part of the benign mesenchymal odontogenic tumors in the 2022 WHO classification. A minority of COsFs are linked to inactivating mutations in the tumor suppressor gene *CDC73* (*HRPT2*), especially in those cases that are part of hyperparathyroidism–jaw tumor syndrome. COsF can also be part of gnathodiaphyseal dysplasia, which is characterized by *GDD1* gene mutations [2, 11].

## Malignant odontogenic tumors

**Fig. 3:**
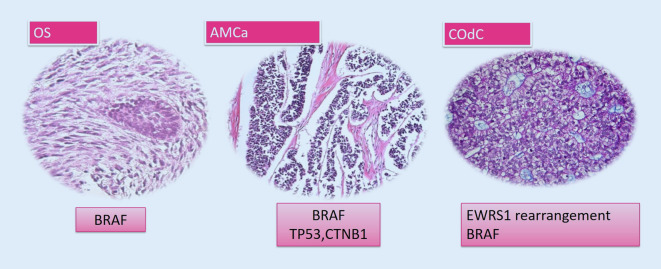
Malignant odontogenic tumors and gene mutations

Malignant odontogenic tumors (MOTs) are extremely rare tumors which arise either de novo or from the malignant transformation of benign odontogenic tumors. They can occur as either carcinomas or sarcomas [1, 5]. In recent studies, malignant odontogenic tumors have also been included in the spectrum of MAPK pathway-driven tumors ([5, 11]; Fig. 3).

### Entities

#### Ameloblastic carcinoma

Ameloblastic carcinoma (AMCa) is a highly aggressive, malignant epithelial odontogenic tumor. It is now defined as an entity which is not related to ameloblastoma [2]. However, AMCas harbor *BRAF* p.V600E mutations like other ameloblastoma-related tumors, with varying prevalence from 25 to 40% [5]. Mutations in other genes which are not related to MAPK/ERK, such as *TP53, CTNNB1*, and *APC*, have also been reported inAMCas [2, 5, 11].

#### Clear cell odontogenic carcinoma

Clear cell odontogenic carcinoma (COdC) is a malignant tumor with high recurrence rate (40%). Its regional lymph node metastases are more common than distant ones and the death rate is about 11%. Differential diagnosis can be critical, which includes jawbone clear cell-containing tumors such as CEOT, amyloid-rich odontogenic fibroma, odontogenic carcinoma with dentinoid, primary or metastatic tumors of salivary glands (e.g., mucoepidermoid carcinoma, clear cell carcinoma, epithelial myoepithelial carcinoma), and metastatic tumors (i.e., clear cell renal carcinoma, melanoma). COdC is characterized by *EWSR1* gene rearrangement in about 80% of cases. It has also been shown to harbor *BRAF* p.V600E in limited cases [2, 5].

#### Odontogenic sarcoma

Odontogenic sarcoma (OS) is a mixed tumor, histologically characterized by a benign ameloblastic epithelium within a sarcomatous mesenchymal component, with or without dentine and enamel. OS can arise de novo or emerge from a sarcomatous change in AF, and approximately one third of AFS (Ameloblastic Fibrosarcoma) cases stem from a recrudescent AF. Clinically, OS shows locally aggressive behavior. The tumor shows a high recurrence rate of approximately 37% [12]. The *BRAF* p.V600E mutation has been detected in 67–71% of reported cases. An *NRAS* mutation has also been reported in one case of OS in a mutually exclusive manner with *BRAF *p.V600E [5, 11]. Although the rarity of this tumor precludes extensive knowledge about its molecular pathology, the current results could support the role of the MAPK/ERK pathway in its pathogenesis and pave the way for further investigations on targeted therapy [5].

## Practical conclusion

- Molecular profiling of *BRAF, SMO, KRAS*, and *bCAT* dissects most odontogenic tumors into three groups.
- Molecular profiling helps to identify patients for neoadjuvant therapies and saves postoperative morbidity.
- A practical approach could be to stain *BRAF* p.V600E by immunohistochemistry and apply other markers dependent on morphology.
- In the rare cases of malignant odontogenic tumors, reference pathology is highly recommended. Be aware of clear cell odontogenic carcinoma.

## Declarations

For this article no studies with human participants or animals were performed by any of the authors. All studies mentioned were in accordance with the ethical standards indicated in each case.

The supplement containing this article is not sponsored by industry.
